# Efficacy and safety of different curcumin formulations in osteoarthritis: an umbrella review of systematic reviews

**DOI:** 10.3389/fmed.2026.1801273

**Published:** 2026-05-21

**Authors:** Chuankai Shi, Taimin Zhang, Yaru Xie, Jiajia Li, Changxu Chen, Jiangwei Liu

**Affiliations:** 1 Department of Graduate School, Xinjiang Medical University, Urumqi, China; 2 Desert Medicine Laboratory, General Hospital of Xinjiang Military Command, Urumqi, China; 3 Xinjiang Medical University, Urumqi, China

**Keywords:** bioavailability, curcumin, osteoarthritis, systematic review, umbrella review

## Abstract

**Background:**

This umbrella review synthesizes evidence from systematic reviews and meta-analyses evaluating the efficacy and safety of diverse curcumin formulations in the treatment of osteoarthritis (OA). The objective is to assess curcumin’s therapeutic potential and inform future formulation development.

**Methods:**

A systematic search was conducted across PubMed, Web of Science, Cochrane, Embase, Scopus, and MEDLINE up to September 2025 to identify systematic reviews and meta-analyses investigating curcumin for OA (requiring at least one randomized controlled trial). Methodological quality was assessed using AMSTAR-2. Due to limited data and substantial heterogeneity in formulations and study designs, findings were synthesized qualitatively.

**Results:**

Ten meta-analyses and systematic reviews were included. Curcumin formulations exhibited significant improvements in pain (VAS) and joint function/stiffness (WOMAC), showing efficacy signals comparable to non-steroidal anti-inflammatory drugs (NSAIDs) and a more favorable tolerability profile in the available studies. However, methodological and clinical heterogeneity was substantial (AMSTAR-2 rated only three studies as high quality). Variations in curcumin composition, bioavailability-enhancing strategies, and extraction methods, coupled with the absence of direct comparative analyses among formulations, precluded definitive inter-group comparisons.

**Conclusion:**

Curcumin-based interventions show promise for OA symptom management, with efficacy signals comparable to NSAIDs and appear to have a more favorable tolerability profile in the available studies, while addressing bioavailability challenges. Nevertheless, pronounced heterogeneity and the lack of head-to-head comparative studies, and incomplete safety reporting across reviews preclude definitive conclusions regarding superiority over NSAIDs or optimal formulations, and formulation-specific inferences remain limited. Future research should prioritize high-quality comparative trials or network meta-analyses to confirm efficacy signals and enable formulation-specific inferences.

**Systematic review registration:**

https://www.crd.york.ac.uk/PROSPERO, identifier CRD420251172722.

## 1 Introduction

Osteoarthritis (OA) represents a leading global cause of joint pain and functional disability, substantially impairing patients’ quality of life (1). The disease pathogenesis encompasses multifaceted mechanisms, including inflammatory cascades, oxidative stress, miRNA and lncRNA dysregulation, alongside genetic and epigenetic alterations (2–4). A characteristic feature of OA involves low-grade chronic inflammation, which perpetuates cartilage degradation, synovitis, and aberrant bone remodeling (5). Recent studies have further elucidated the critical roles of diverse immune cell populations within the bone-joint microenvironment, highlighting their contribution to the pathogenesis of musculoskeletal disorders (6, 7).

Amidst accelerating population aging, the disease burden of OA continues to rise. Currently, no disease-modifying therapies exist, and clinical management predominantly targets symptomatic relief (8). Non-steroidal anti-inflammatory drugs (NSAIDs), as first-line pharmacological agents, provide symptomatic improvement but are associated with gastrointestinal bleeding and cardiovascular risks upon prolonged administration. Systematic reviews and meta-analyses of observational studies indicate substantial heterogeneity in NSAID utilization among OA patients, with generally limited evidence quality (9). Although opioids demonstrate analgesic efficacy, their addictive potential and adverse effects contribute to high discontinuation rates in OA treatment, as evidenced by meta-analytical data (10). Surgical approaches such as joint replacement remain reserved for end-stage disease; however, procedural risks persist, and a subset of patients report refractory pain and functional deficits postoperatively (11). These therapeutic limitations highlight the critical demand for novel, safe, and efficacious OA treatment strategies.

Curcumin, a bioactive polyphenolic compound derived from *Curcuma longa*, exhibits a pleiotropic mechanism of action in OA management, encompassing suppression of pro-inflammatory cytokines, modulation of matrix metalloproteinase activity, and attenuation of oxidative stress (12). Emerging evidence has identified histone lactylation—a lactate-driven epigenetic modification—as a key player in OA progression, suggesting that targeting lactate metabolism or lactylation pathways may offer new therapeutic opportunities (13). Its therapeutic profile is characterized by superior safety and tolerability relative to conventional NSAIDs, with a notably reduced incidence of adverse effects (14). Nevertheless, the clinical translation of native curcumin is constrained by intrinsic pharmacokinetic limitations, including poor aqueous solubility, limited systemic bioavailability following oral administration, and rapid metabolic clearance (15). To address these challenges, multiple formulation strategies have been developed, ranging from conventional preparation optimization to advanced drug delivery systems and innovative manufacturing technologies (16).

The burgeoning development of diverse curcumin formulations has introduced substantial heterogeneity into clinical evidence systems. Although the therapeutic potential of curcumin for OA has been well-documented, a pivotal question persists: whether novel curcumin-based formulations exhibit superior clinical efficacy relative to conventional curcumin preparations. The existing literature remains notably fragmented, with individual randomized controlled trials (RCTs) and meta-analyses predominantly examining isolated formulations or heterogeneous mixtures, while failing to provide rigorous comparative assessments across distinct formulation types. Consequently, the field lacks a robust comparative evidence framework, precluding evidence-based prioritization among available formulations.

Given the paucity of meta-analyses comparing different formulation types, this umbrella review employs a qualitative synthesis and critical appraisal approach, extending beyond traditional quantitative comparisons. Our primary objective is to systematically evaluate existing curcumin formulations, synthesize comprehensive evidence pertaining to OA, and offer strategic insights to guide future advancements in this domain.

The optimization of conventional curcumin formulations primarily aims to enhance bioavailability through the incorporation of excipients and formulation modifications. Excipients including milk constituents, sugars, milk fat, and piperine from black pepper have been demonstrated to significantly improve curcumin solubility and bioavailability (17). Solid dispersion technology has emerged as another effective strategy, with curcumin solid dispersions prepared using d-α-tocopherol polyethylene glycol 1000 succinate (TPGS) and mannitol showing substantial improvements in solubility and dissolution profiles, achieving 90% drug release within 10 min (18). Furthermore, cyclodextrin inclusion complexation enhances curcumin’s aqueous solubility and intestinal absorption through molecular encapsulation (19). Nanocarrier systems represent the most advanced approach in curcumin formulation development, where particle size reduction and surface modification significantly improve physicochemical properties and pharmacokinetic behavior, thereby enhancing solubility, stability, and bioavailability (20, 21). Although primarily investigated in osteolysis models, recent nanomedicine studies have demonstrated that advanced nanocarrier strategies can effectively modulate oxidative stress and enable targeted gene delivery—insights that may inform the design of improved curcumin nanoformulations for OA (22). Recent innovations in formulation technologies, including microsphere encapsulation and transdermal delivery systems (23, 24), have not only optimized administration routes but also expanded therapeutic applications in OA management.

Based on these considerations, our study classifies curcumin preparations into three distinct categories: (i) Conventional curcumin preparations, comprising standard curcumin extracts or analogues that do not explicitly incorporate bioavailability-enhancing technologies; (ii) Bioavailability-enhanced curcumin preparations, characterized by the explicit use of specific technologies to improve absorption, such as phospholipid complexes, nano-formulations, piperine combinations, micellization, or liposomal formulations; and (iii) Whole turmeric extracts/non-standardized extracts, derived from the complete turmeric rhizome where the curcuminoid content is not standardized or is utilized in its whole form.

Through systematic evidence classification and synthesis, this study seeks to delineate the current research landscape, identify critical knowledge gaps, establish priorities for future investigations, and ultimately provide clinically relevant insights for optimizing OA therapeutic strategies.

## 2 Methods

### 2.1 Design and registration

As part of this umbrella meta-analysis, we followed the Preferred Reporting Items for Systematic Reviews and Meta-analysis (PRISMA) guidelines (25). A PROSPERO registration was made for the study protocol (Registration code: CRD420251172722).

### 2.2 Eligibility criteria

This study incorporated systematic reviews and meta-analyses examining the therapeutic efficacy and/or safety profile of curcumin-based interventions for OA in human subjects. Eligible studies were required to synthesize data exclusively from RCTs. The inclusion criteria encompassed reviews comparing curcumin (regardless of formulation, dosage regimen, or treatment duration) with placebo controls, active comparators (including NSAIDs and glucosamine), or different curcumin formulations. Studies were excluded if they evaluated combination therapies where the specific effects of curcumin could not be isolated.

### 2.3 Population

The study population comprised individuals clinically diagnosed with osteoarthritis affecting any joint, with no exclusion criteria applied based on age, sex, or ethnic background.

### 2.4 Intervention

The intervention was characterized as the oral administration of any curcumin-based formulation employed as monotherapy. This encompassed three predetermined classifications: (i) Conventional curcumin formulations, consisting of standard curcumin extracts or analogues that do not explicitly incorporate bioavailability-enhancing technology; (ii) Bioavailability-enhanced curcumin formulations, characterized by the explicit use of specific technologies to improve bioavailability, such as phospholipid complexes, nano-formulations, piperine combinations, micellization, or liposomal formulations; and (iii) Whole turmeric extracts or non-standardized extracts, derived from the complete turmeric rhizome where the curcuminoid content is not standardized or is utilized in its whole form.

This tripartite classification was based on two primary criteria derived from formulation science and clinical reporting standards: the explicit use of bioavailability-enhancing technologies, and the degree of standardization of curcuminoid content. Category 1 serves as the baseline comparator, representing conventional extracts without bioavailability enhancement. Category 2 includes formulations with explicitly stated technologies (e.g., piperine co-administration, phospholipid complexes, nanoparticles, micelles) that have pharmacokinetic evidence of improved absorption. Category 3 reflects real-world usage of whole turmeric or non-standardized extracts, where curcuminoid content is not quantified, acknowledging their presence in traditional medicine and certain clinical trials while recognizing their inherent batch-to-batch variability. To ensure transparent and reproducible assignment, especially for mixed or poorly described products, a sequential decision algorithm was applied by two independent reviewers (with a third arbiter): (i) if described as whole or non-standardized turmeric extract without curcuminoid quantification → Category 3; (ii) if any bioavailability-enhancing technology is explicitly reported → Category 2, even if combined with other components; (iii) if described only as curcumin, curcuminoids, or a standardized extract without such technology → Category 1; (iv) if insufficiently described (e.g., only “curcumin extract”) and unassignable after full-text review → flagged as “unclassifiable.” Categorization was applied *post hoc* based on intervention descriptions, with no restrictions on dosage, treatment duration, or dosing frequency, aiming to capture the full evidence scope; all variations were documented as potential heterogeneity sources.

### 2.5 Study designs

This study exclusively incorporated systematic reviews and meta-analyses, while narrative reviews, scoping reviews, study protocols, conference abstracts, and individual RCTs were systematically excluded from the analysis.

### 2.6 Information sources

A systematic literature search was conducted across six major electronic databases (PubMed, Web of Science, Cochrane, Embase, Scopus, and MEDLINE) from their inception through September 2025. To ensure comprehensive coverage, manual screening of reference lists from all included review articles was performed to identify additional potentially relevant studies that might not have been retrieved through the primary database search. Gray literature was not systematically searched, which is acknowledged as a potential limitation of this review.

### 2.7 Search strategy

The search strategy was systematically developed by integrating Medical Subject Headings (MeSH) terms and free-text keywords encompassing three principal domains: (i) Osteoarthritis (e.g., “osteoarthritis,” “degenerative joint disease”); (ii) Curcumin (e.g., “curcumin,” “Curcuma longa extract,” “diferuloylmethane”); and (iii) Study design (e.g., “systematic review,” “meta-analysis”). Detailed search syntax tailored to each database is documented in the Supplementary materials. While no temporal or linguistic restrictions were imposed during the initial database searches, only full-text articles published in English were considered for final inclusion.

### 2.8 Study selection

The identified records were imported into EndNote 21 for duplicate removal. Two independent reviewers conducted the study selection process in accordance with predefined inclusion criteria. Initial screening was performed at the title and abstract level, followed by comprehensive evaluation of full-text articles deemed potentially eligible. Discrepancies between reviewers were resolved through consensus or, when necessary, by arbitration with a third senior investigator.

### 2.9 Assessment of methodological quality

The methodological quality of the included systematic reviews and meta-analyses was independently evaluated by two investigators utilizing the Assessment of Multiple Systematic Reviews 2 (AMSTAR 2) instrument (26). The AMSTAR 2 checklist comprises 16 domains, with responses categorized as “Yes,” “Partial Yes,” or “No.”

In accordance with the tool’s recommendations, no composite score was calculated. Instead, the confidence in the findings of each study was classified as “High,” “Moderate,” “Low,” or “Critically Low,” contingent upon the identification of critical and non-critical methodological limitations: “High” confidence indicates no critical flaws (or at most one non-critical weakness), “Moderate” indicates more than one non-critical weakness, “Low” indicates one critical flaw (with or without non-critical weaknesses), and “Critically low” indicates more than one critical flaw. Discrepancies between the two assessors were resolved through consensus or, when necessary, by adjudication from a third reviewer.

### 2.10 Data extraction

Two independent reviewers systematically extracted data from eligible studies using a standardized, pilot-tested extraction form. The extracted data encompassed: first author’s name and publication year; study characteristics including the number and design of included primary studies; total sample size; demographic and clinical characteristics of participants (e.g., affected osteoarthritis joint); intervention details (curcumin formulation, dosage, and administration route) and comparator treatments; duration of intervention; outcome measures (including specific assessment scales); and quantitative results (presented as pooled mean differences, standardized mean differences, odds ratios, or risk ratios with corresponding 95% confidence intervals). Additionally, data regarding study heterogeneity (quantified by I^2^ statistics) and publication bias assessments were extracted when available in the original studies.

### 2.11 Data summary

Given the considerable clinical and methodological heterogeneity observed among the included meta-analyses—particularly with respect to variations in curcumin formulations, dosages, and study populations—conducting a quantitative meta-analysis of pooled effect estimates was considered methodologically unsound. Consequently, a narrative synthesis approach was employed. The findings are presented descriptively, categorized by outcome (efficacy: VAS, WOMAC subscales; safety: adverse events) and comparator (placebo versus active control). The results are systematically summarized in structured tables, and the overall robustness and consistency of the evidence are qualitatively assessed and discussed.

### 2.12 Assessment of overlap of primary studies

To evaluate the degree of overlap of primary RCTs across the included systematic reviews, we constructed a citation matrix of all unique primary studies. For each included systematic review, we extracted the list of primary RCTs as reported in the original articles (typically from tables or reference lists). A binary matrix was created with rows representing unique RCTs and columns representing the 10 systematic reviews; a cell was marked as “1” if the RCT was included in that review and “0” otherwise. The total number of citations (N) was calculated as the sum of all “1” entries. The number of unique RCTs (r) was the row count. The number of systematic reviews (c) was 10. The corrected covered area (CCA) was calculated using Equation 1:


$$C\,C\,A=\frac{N-r}{r\times c-r}$$ (1)

CCA values were interpreted as follows: 0%–5% = no overlap, 6%–10% = slight overlap, 11%–15% = moderate overlap, >15% = high overlap (27).

## 3 Results

### 3.1 Literature search results

**FIGURE 1:**
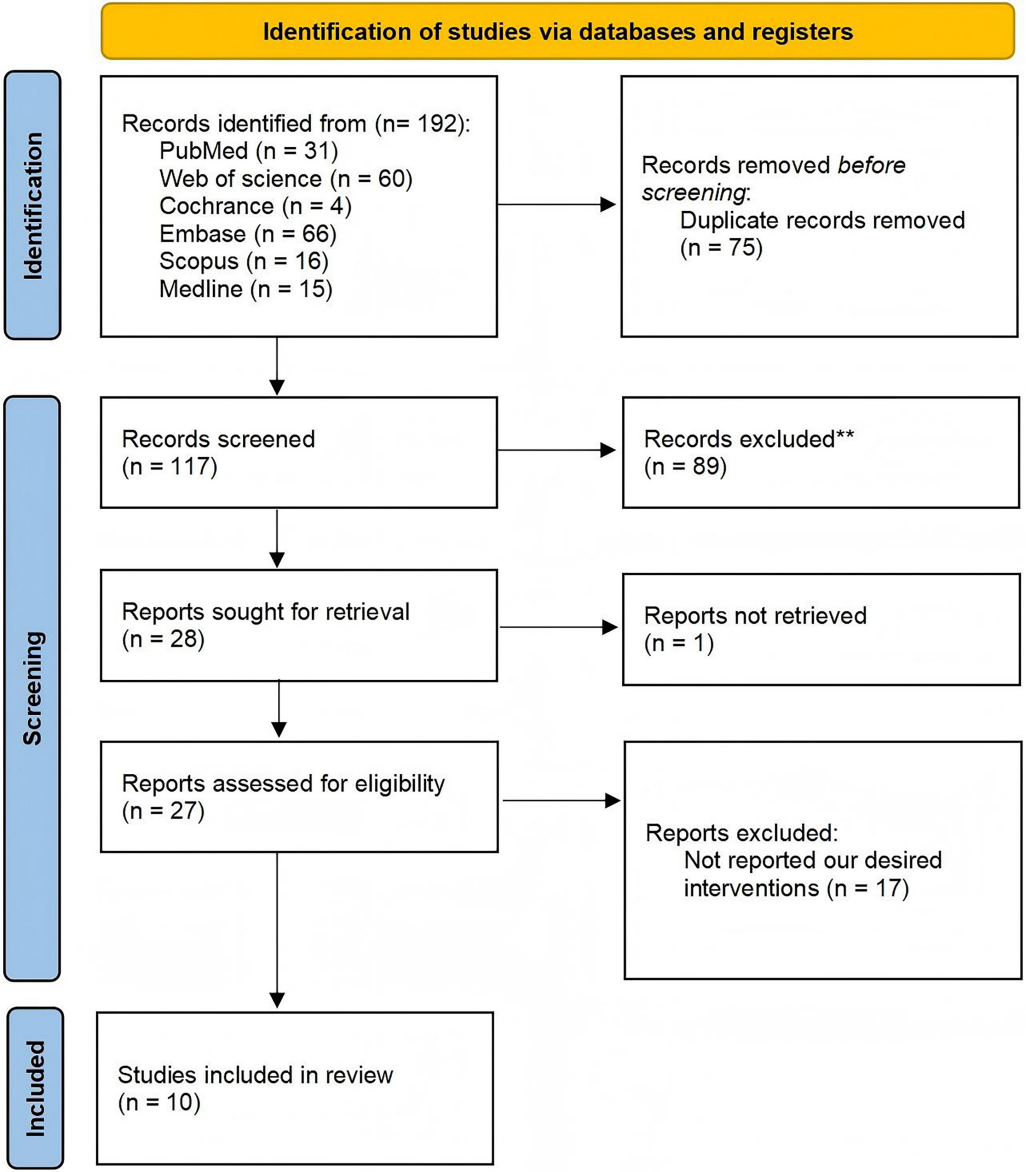
PRISMA flowchart for the inclusion of studies.

A comprehensive search of electronic databases yielded 192 relevant articles. Following the removal of 75 duplicate records, 117 publications underwent initial screening based on titles and abstracts. Subsequently, 27 full-text articles were assessed for eligibility, from which 10 studies met the inclusion criteria and were selected for final analysis. The study selection process is detailed in the PRISMA flowchart (Figure 1).

### 3.2 Basic characteristics of studies

The included studies, published between 2017 and 2025, consisted of four clinical trials conducted in China (28–31), as well as individual studies from Indonesia (32), Iran (33), Spain (34), Thailand (35), the United Kingdom (36), and the United States (37). All enrolled participants had been clinically diagnosed with OA or KOA. A detailed overview of the study characteristics is provided in Table 1 (28–37). The therapeutic interventions covered all categories specified in this systematic analysis, including Curcumin C3 Complex^®^, curcuminoids, Theracurmin^®^, BCM-95^®^, and Curcuma longa extract. Comparator groups received either placebo, NSAIDs (e.g., ibuprofen and diclofenac), or glucosamine. All studies reported outcome measures that aligned with the parameters evaluated in the present investigation. Furthermore, while a minority of studies received government funding, the majority did not report any financial support.

**TABLE 1:** Study characteristic of included studies.

References	Location	Included studies	Health condition	Age and gender	Intervention	Control	Outcomes	Funding	Searches
Igho et al. (36)	UK	7 (*n* = 797)	KOA	57–69 M/F	Category 1 + 2 + 3	Placebo and Ibuprofen	(Primary) pain, physical function and quality of life (Secondary) use of rescue medication, adverse events and drop-out rates	None	Inception till March 2015 (updated in November 2016)
Paultre et al. (37)	USA	10 (*n* = 1,287)	KOA	56.85 ± 9.19 M/F	Category 1 + 2	Placebo, NSAIDs and Glucosamine	Mean baseline and follow-up WOMAC or VAS	None	Inception till 2019
Shokri-Mashhadi et al. (33)	Iran	17 (*n* = 1,758)	KOA	38–80 M/F	Category 1 + 2 + 3	Usual treatment, placebo, NSAIDs	(Primary) the improvement of WOMAC scores and VAS (Secondary) evaluation of changes in other pains and performance, knee injury questionnaires	NA	Inception till 2020
Zeng et al. (30)	China	15 (*n* = 1,621)	OA	NR M/F	Category 1 + 3	Placebo, NSAIDs and glucosamine	(Primary) pain, joint function, joint stiffness, and adverse events (Secondary) other assessments score of OA [such as the knee injury and osteoarthritis score (KOOS)] and biochemical indicators	None	Inception till 2020
Feng et al. (28)	China	15 (*n* = 6,70)	KOA	NR M/F	Category 1 + 2 + 3	Placebo and NSAIDs	(Primary) VAS for pain, WOMAC pain score, WOMAC function scores, and adverse events (Secondary) other outcomes	Government	Inception till 2022
Gutiérrez Ruiz et al. (34)	Spain	5 (*n* = 1,186)	KOA	40–80 M/F	Category 1 + 3	Placebo and NSAIDs	Describing changes in VAS, WOMAC, JKOM and KOOS indexes and sColl2-1, ROS and IL-1B markers	None	2010–2020
Wan et al. (29)	China	14 (*n* = 1,533)	KOA	NR M/F	Category 1 + 2 + 3	NSAIDs, chondroitin sulfate and glucosamine	Clinical improvement and safety	NA	Inception till 2021
Zhao et al. (31)	China	23 (*n* = 2,175)	KOA	NR M/F	Category 1 + 2 + 3	Placebo and NSAIDs	VAS, WOMAC, use of rescue medication and adverse events	Government	Inception till 2023
Hidayat et al. (32)	Indonesia	10 (*N* = 786)	KOA	NR M/F	Category 3	Placebo and NSAIDs	VAS and WOMAC	None	2013–2023
Wai et al. (35)	Thailand	17 (*n* = 1,736)	KOA	NR M/F	Category 1 + 2	Placebo and NSAIDs	(Primary) WOMAC (Secondary) the change from baseline of each subscale of WOMAC, changes in other pain scores and adverse events	None	Inception till 2024

### 3.3 Methodological quality evaluation

The methodological quality of all 10 articles included in this review was systematically evaluated using the AMSTAR 2 tool. As detailed in Table 2 (28–37), quality assessment results demonstrated that three studies were classified as high-quality, 1 as moderate, 4 as low, and 2 as very low.

**TABLE 2:** Use of the AMSTAR-2 Tool in meta-analyses.

References	Q1*	Q2	Q3	Q4	Q5	Q6	Q7	Q8	Q9	Q10	Q11	Q12	Q13	Q14	Q15	Q16	Quality assessment
Igho et al. (36)	Yes	Partial Yes	Yes	Partial Yes	Yes	Yes	Partial Yes	Yes	Yes	Yes	Yes	Yes	Yes	No	No	Yes	Low quality
Paultre et al. (37)	Yes	Yes	Yes	Partial Yes	Yes	Yes	Yes	Yes	Yes	No	NA	NA	Yes	No	NA	Yes	Low quality
Shokri-Mashhadi. et al. (33)	Yes	Partial Yes	Yes	Partial Yes	No	No	Yes	Yes	Yes	No	NA	NA	No	No	NA	No	Very Low quality
Zeng et al. (30)	Yes	Yes	Yes	Partial Yes	Yes	Yes	Partial Yes	Yes	Yes	No	Yes	Yes	Yes	Yes	Yes	Yes	High quality
Feng et al. (28)	Yes	Yes	Yes	Partial Yes	Yes	Yes	Partial Yes	Partial Yes	Yes	Yes	Yes	Yes	Yes	Yes	Yes	Yes	High quality
Gutiérrez Ruiz. et al. (34)	Yes	Yes	Yes	Partial Yes	No	No	Partial Yes	Yes	Yes	No	NA	NA	Yes	No	NA	Yes	Very Low quality
Wan et al. (29)	Yes	Yes	Yes	Partial Yes	Yes	Yes	Yes	Yes	Yes	No	Yes	Yes	Yes	No	No	Yes	Low quality
Zhao et al. (31)	Yes	Yes	Yes	Partial Yes	Yes	Yes	Yes	Yes	Yes	No	Yes	Yes	Yes	No	Yes	Yes	Moderate quality
Hidayat et al. (32)	Yes	Yes	Yes	Partial Yes	Yes	Yes	Partial Yes	Yes	Yes	No	Yes	Yes	Yes	Yes	Yes	Yes	High quality
Wai et al. (35)	Yes	Yes	Yes	Yes	Yes	Yes	Yes	Yes	Yes	No	Yes	Yes	Yes	No	No	Yes	Low quality

NA: not applicable. *1. Did the research questions and inclusion criteria for the review include the components of PICO? 2. Did the report of the review contain an explicit statement that the review methods were established prior to the conduct of the review and did the report justify any significant deviations from the protocol? 3. Did the review authors explain their selection of the study designs for inclusion in the review? 4. Did the review authors use a comprehensive literature search strategy? 5. Did the review authors perform study selection in duplicate? 6. Did the review authors perform data extraction in duplicate? 7. Did the review authors provide a list of excluded studies and justify the exclusions? 8. Did the review authors describe the included studies in adequate detail? 9. Did the review authors use a satisfactory technique for assessing the risk of bias (RoB) in individual studies that were included in the review? 10. Did the review authors report on the sources of funding for the studies included in the review? 11. If meta-analysis was performed, did the review authors use appropriate methods for statistical combination of results? 12. If meta-analysis was performed, did the review authors assess the potential impact of RoB in individual studies on the results of the meta-analysis or other evidence synthesis? 13. Did the review authors account for RoB in individual studies when interpreting/ discussing the results of the review? 14. Did the review authors provide a satisfactory explanation for, and discussion of, any heterogeneity observed in the results of the review? 15. If they performed quantitative synthesis, did the review authors carry out an adequate investigation of publication bias (small study bias) and discuss its likely impact on the results of the review? 16. Did the review authors report any potential sources of conflict of interest, including any funding they received for conducting the review? Each question was answered with “Yes”, “Partial Yes,” or “No”. When no meta-analysis was done, question 11, 12, and 15 were answered with “No meta-analysis conducted.

**FIGURE 2:**
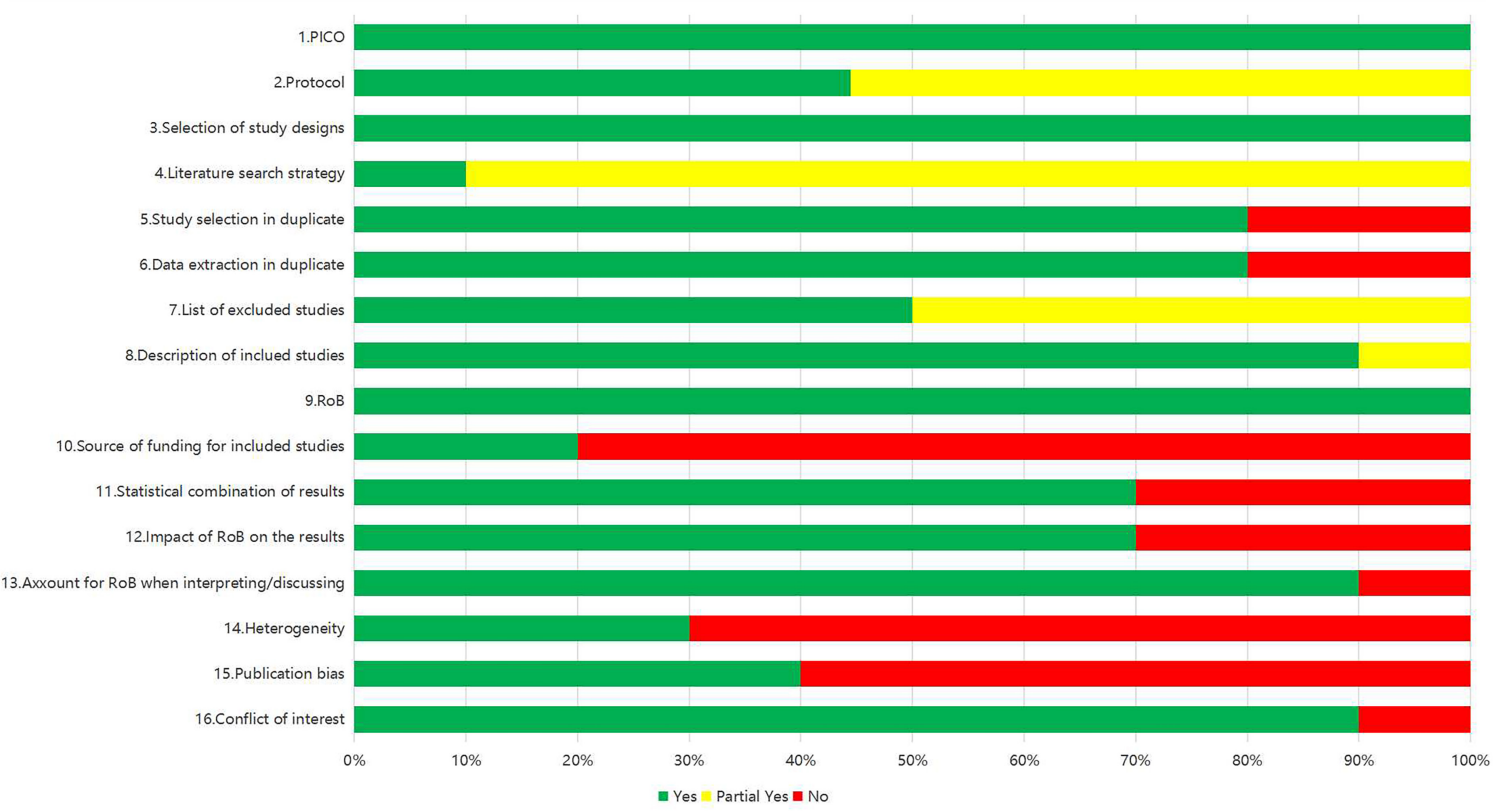
Aggregated AMSTAR-2 ratings for included meta-analyses.

A comprehensive evaluation of the included articles is presented in Figure 2. All studies exhibited appropriate PICO framework implementation and adequate risk of bias assessment. However, several methodological limitations were identified: failure to report funding sources (*n* = 8; 80%), insufficient investigation of heterogeneity sources (*n* = 7; 70%), and lack of discussion on potential publication bias (*n* = 6; 60%). Positive evaluations were assigned for study protocol registration, appropriate research design selection, comprehensive search strategies, well-justified exclusion criteria, and detailed descriptions of included studies. Screening and data extraction were independently performed by two reviewers for eight studies (80%).

Given the inclusion of three systematic reviews in this analysis, questions 11, 12, and 15 of the AMSTAR 2 assessment were deemed not applicable (NA). Among the remaining seven studies, appropriate meta-analytic methodology and evaluation of bias risk impact on results were consistently reported. Notably, 90% of studies (*n* = 9) explicitly disclosed potential conflicts of interest.

### 3.4 Evidence synthesis for the primary outcome

#### 3.4.1 Therapeutic effect: the overall effect of curcumin on pain and function

Table 3 (28–32, 35, 36) and Table 4 (28–32, 35–37) present a synthesis of findings from seven meta-analyses examining the association between curcumin supplementation and OA. The results are stratified by outcome measures, beginning with pain assessment via the VAS, followed by evaluation using the WOMAC and its subscales.

**TABLE 3:** Outcomes of meta-analysis: VAS.

References	Control	*k*	Estimate	95%CI	*P*-value	I^2^	Publication bias^a^
Igho et al. (36)	Placebo	5	SMD = −3.30	−4.99 to −2.01	<0.00001	95%	NR
Zeng et al. (30)	Placebo	6	WMD = −11.55	−14.3 to −9.06	<0.00001	69%	*P* = 0.125 (E)
	NSAIDs	2	WMD = −0.34	−1.25 to 0.57	0.46	0%	*P* = 0.125 (E)
Feng et al. (28)	Placebo	9	WMD = −1.77	−2.44 to −1.09	<0.001	86.8%	*P* = 0.318 (E) Susp. (F)
		8	WMD = −1.36	−1.76 to −0.97	<0.001	56.9%	*P* = 0.318 (E) Susp. (F)
	NSAIDs	3	WMD = −0.3	−0.63 to 0.04	0.082	6.3%	*P* = 0.318 (E) Susp. (F)
Wan et al. (29)	NSAIDs	4	SMD = 0.21	−0.02 to 0.43	0.08	0%	NR
	Chondroitin sulfate combined with glucosamine	2	SMD = 4.21	1.58 to 6.84	<0.0001	95%	NR
	Glucosamine sulfate	1	SMD = 3.16	2.18 to 4.13	NR	NR	NR
Zhao et al. (31)	Placebo	9	MD = −1.63	−2.91 to −0.45	NR	NR	Susp. (F)
Hidayat et al. (32)	Placebo	6	MD = 18.25	7.79 to 28.72	0.0006	99%	Susp. (F)
Wai et al. (35)	Active drug comparator	1	MD = 0.00	−2.38 to 2.38	NR	0%	NR
	Placebo	3	MD = −16.77	−20.94 to −12.60	NR	3.3%	NR

^a^Publication bias: NS indicates not significant; (E) indicates Egger’s test. Susp. (F) indicates suspected publication bias based on funnel plot inspection; Not Susp. (F) indicates that publication bias is not suspected based on funnel plot inspection; NR indicates not reported. Underlined values are significant. Underlined values are statistically significant, and all such values (whether positive or negative) indicate that curcumin is more effective than the comparator. The sign (positive vs. negative) varies depending on how different meta-analyses calculated effect sizes and which comparator was used.

**TABLE 4:** Outcomes of meta-analysis: WOMAC.

References	Control	*k*	Estimate	95% CI	*P*-value	I^2^	Publication bias^a^
WOMAC pain score							
Igho et al. (36)	Control	3	SMD = −0.80	−2.08 to 0.48	0.22	97%	NR
	Placebo	2	MD = −1.58	−3.75 to 0.59	0.15	NR	NR
	Ibuprofen	1	MD = 0.18	0.12 to 0.24	<0.0001	NR	NR
Zeng et al. (30)	Placebo	4	SMD = −0.66	−0.88 to −0.43	<0.0001	34%	*P* = 0.301 (E)
	NSAIDs	1	SMD = 0.04	−0.18 to 0.25	0.72	NR	*p* = 0.301 (E)
Feng et al. (28)	Placebo	6	WMD = −1.94	−2.91 to −0.97	<0.001	72.9%	*P* = 0.78 (E) Susp. (F)
		5	WMD = −2.28	−3.05 to −1.52	<0.001	51.8%	*P* = 0.78 (E) Susp. (F)
	NSAIDs	2	WMD = 0.24	−0.47 to 0.96	0.505	0.0%	*P* = 0.78 (E) Susp. (F)
Wan et al. (29)	NSAIDs	2	SMD = −0.06	−0.24 to 0.12	0.54	0%	NR
	Chondroitin sulfate combined with glucosamine	2	SMD = 0.82	−0.54 to 2.18	0.24	94%	NR
Zhao et al. (31)	Placebo	8	MD = −2.24	−4.57 to −0.52	NR	NR	Susp. (F)
Wai et al. (35)	Active drug comparator	1	MD = −0.86	−2.33 to 0.61	NR	0.0%	NR
	Placebo	4	MD = −2.47	−3.25 to −1.68	NR	35.9%	NR
WOMAC function score							
Igho et al. (36)	Control	3	SMD = −1.84	−3.54 to −0.13	0.04	98%	NR
	Placebo	2	MD = −2.56	−3.29 to −1.83	<0.00001	NR	NR
	Ibuprofen	1	MD = −0.05	−0.09 to −0.01	0.02	NR	NR
Zeng et al. (30)	Placebo	4	SMD = −0.79	−1.27 to −0.31	=0.001	75%	*P* = 0.565 (E)
	NSAIDs	1	SMD = 0.07	−0.14 to 0.29	0.51	NR	*P* = 0.565 (E)
Feng et al. (28)	Placebo	6	WMD = −6.36	−8.94 to −3.78	<0.001	79.2%	*P* = 0.515 (E) Susp. (F)
		5	WMD = −7.21	−9.71 to −4.72	<0.001	70.7%	*P* = 0.515 (E) Susp. (F)
	NSAIDs	2	WMD = −0.57	−3.07 to 1.94	0.657	0.0%	*P* = 0.515 (E) Susp. (F)
Wan et al. (29)	NSAIDs	3	SMD = 0.04	−0.14 to 0.22	0.65	0%	NR
	Chondroitin sulfate combined with glucosamine	2	SMD = 2.92	2.03 to 3.81	<0.00001	72%	NR
Zhao et al. (31)	Placebo	7	MD = −0.753	−1.59 to 0.0611	NR	NR	Susp(F)
Wai et al. (35)	Active drug comparator	1	MD = −4.81	−9.73 to 0.11	NR	0.0%	NR
	Placebo	3	MD = −9.62	−12.47 to −6.76	NR	38.3%	NR
WOMAC stiffness score							
Igho et al. (36)	Control	3	SMD = 0.10	−1.08 to 1.29	0.86	97%	NR
	Placebo	2	MD = −0.30	−0.47 to −0.13	0.0004	4%	NR
	Ibuprofen	1	MD = 0.22	0.17 to 0.27	<0.00001	NR	NR
Zeng et al. (30)	Placebo	4	SMD = −0.35	−0.57 to −0.12	0.002	26%	*P* = 0.138 (E)
	NSAIDs	1	SMD = 0.05	−0.17 to 0.27	0.65	NR	*P* = 0.138 (E)
Feng et al. (28)	Placebo	6	WMD = −0.54	−1.03 to −0.05	0.031	77.6%	*P* = 0.63 (E) Susp(F)
		5	WMD = −0.31	−0.56 to −0.05	0.018	0.0%	*P* = 0.63 (E) Susp. (F)
	NSAIDs	2	WMD = 0.19	−0.17 to 0.56	0.298	0.0%	*P* = 0.63 (E) Susp. (F)
Wan et al. (29)	NSAIDs	2	SMD = −0.10	−0.28 to 0.08	0.29	0%	NR
	Chondroitin sulfate combined with glucosamine	2	SMD = 0.45	0.13 to 0.77	0.006	0%	NR
Zhao et al. (31)	Placebo	7	MD = −8.83	−21.9 to 2.88	NR	NR	Susp. (F)
Wai et al. (35)	Active drug comparator	1	MD = 0.60	−0.03 to 1.23	NR	0.0%	NR
	Placebo	3	MD = −0.78	−1.58 to 0.03	NR	88.5%	NR
WOMAC total score							
Igho et al. (36)	Control	4	SMD = −3.29	−6.23 to −0.35	0.03	99%	NR
	Placebo	3	SMD = −4.42	−6.66 to −2.19	0.0001	93%	NR
	Ibuprofen	1	MD = −0.03	−0.03 to 0.09	0.29	NR	NR
Feng et al. (28)	Placebo	4	WMD = −10.47	−15.65 to −5.3	<0.001	80.6%	*P* = 0.96 (E) Susp. (F)
		3	WMD = −12.88	−14.79 to −10.98	<0.001	0.0%	*P* = 0.96 (E) Susp. (F)
	NSAIDs	3	WMD = −0.68	−3.88 to 2.52	0.676	80.6%	*P* = 0.96 (E) Susp. (F)
Wan et al. (29)	NSAIDs	3	SMD = 0.03	−0.14 to 0.21	0.70	0%	NR
	Chondroitin sulfate combined with glucosamine	2	SMD = 2.39	1.71 to 3.07	<0.00001	60%	NR
	Glucosamine sulfate	1	SMD = 18.50	19.1 to 25.09	NR	NR	NR
Zhao et al. (31)	Placebo	9	MD = −18.89	−29.53 to −8.76	NR	NR	Susp. (F)
Hidayat et al. (32)	Placebo	2	MD = 8.12	−2.11 to 18.35	0.12	98%	Susp. (F)
	NSAIDs	2	MD = −11.99	−39.21 to 15.23	0.39	80%	Susp. (F)

^a^Publication bias: NS indicates not significant; (E) indicates Egger’s test. Susp. (F) indicates suspected publication bias based on funnel plot inspection; Not Susp. (F) indicates that publication bias is not suspected based on funnel plot inspection; NR indicates not reported. Underlined values are significant. Underlined values are statistically significant, and all such values (whether positive or negative) indicate that curcumin is more effective than the comparator. The sign (positive vs. negative) varies depending on how different meta-analyses calculated effect sizes and which comparator was used.

All seven meta-analyses reported pooled effect estimates for VAS scores, demonstrating statistically significant reductions in pain with curcumin-based interventions compared to placebo, chondroitin sulfate alone, or combined chondroitin sulfate-glucosamine therapy. However, Hidayat et al. (32) reported a contradictory finding (MD = 18.5; 95% CI: 7.79–28.72). This positive MD reflects that their analysis pooled change-from-baseline scores (improvement values) rather than endpoint scores; a positive value therefore still indicates greater pain reduction with curcumin. The discrepancy in direction is due to different effect size conventions, not contradictory clinical conclusions. Substantial heterogeneity was observed across trials (I^2^ = 99%). This heterogeneity can be classified into three types: clinical heterogeneity (e.g., variations in OA severity, affected joint sites, baseline pain levels, and concurrent illnesses), methodological heterogeneity (e.g., differences in blinding procedures, allocation concealment, outcome assessment tools, and placebo compositions), and statistical heterogeneity (e.g., variations in curcumin formulations, dosages ranging from 450 to 2,000 mg daily, and treatment durations of 5–11 weeks). Curcumin demonstrated non-inferior efficacy to NSAIDs, with most studies reporting no statistically significant differences in VAS scores.

For WOMAC outcomes, curcumin formulations showed significant improvements relative to placebo across pain, function, stiffness, and total scores. Compared with NSAIDs, most studies found no significant differences. Wan et al. (29) reported no significant difference in WOMAC pain between curcumin and chondroitin sulfate/glucosamine (SMD = 0.82; 95% CI: −0.54 to 2.18), but curcumin showed superior outcomes in function (SMD = 2.92; 95% CI: 2.03 to 3.81), stiffness reduction (SMD = 0.45; 95% CI: 0.13 to 0.77), and overall WOMAC scores (SMD = 2.39; 95% CI: 1.71 to 3.07).

Potential publication bias was identified by Feng et al. (28), Zhao et al. (31) in funnel plot analyses.

#### 3.4.2 Safety: adverse event analysis

The safety profile of curcumin formulations was systematically evaluated through qualitative synthesis of adverse event data derived from included systematic reviews. Ten meta-analyses reporting adverse events were identified, with outcomes comprehensively summarized in Table 5 (28–37). The most frequently reported adverse events were gastrointestinal in nature, including abdominal pain, diarrhea, dyspepsia, nausea, and bloating. Less common adverse effects encompassed neurological, dermatological, cardiovascular, and metabolic manifestations.

**TABLE 5:** Outcomes of meta-analysis: adverse events.

References	Control	*k*	Estimate	95% CI	*P*-value	I^2^	Publication bias^a^	Adverse events
Igho et al. (36)	Control	5	RR = 0.84	0.66 to 1.08	0.17	0%	NR	Intestinal symptoms/abdominal pain/distension
	Placebo	3	RR = 1.26	0.56 to 2.84	0.57	0%	NR	
	Ibuprofen	2	RR = 0.81	0.63 to 1.05	0.11	0%	NR	
Paultre et al. (37)	NR	29	NR	NR	NR	NR	NR	Gastrointestinal discomfort such as nausea/diarrhea/dyspepsia
Shokri-Mashhadi et al. (33)	NR	NR	NR	NR	NR	NR	NR	Gastrointestinal discomfort
Zeng et al. (30)	Placebo	9	RR = 1.18	0.71 to 1.94	0.52	25%	NS (E)	Diarrhea
	NSAIDs	3	RR = 0.55	0.34 to 0.88	0.01	70%	NS (E)	
Feng et al. (28)	Placebo	9	RR = 1.07	0.70 to 1.65	0.745	32.6%	*P* = 0.179 (E) Susp. (F)	Dyspepsia, diarrhea, nausea and/or constipation, meteorism, stomach pain and gastroesophageal reflux
	NSAIDs	5	RR = 0.65	0.41 to 1.03	0.065	55.8%	*P* = 0.179 (E) Susp. (F)	
		4	RR = 0.63	0.41 to 0.95	0.026	53.7%	*P* = 0.179 (E) Susp. (F)	
Gutiérrez Ruiz et al. (34)	NR	5	NR	NR	NR	NR	NR	Bloating, abdominal pain, nausea, dyspepsia, hypertension, itchy tongue, headache, reflux, diarrhea and digestive discomfort
Wan et al. (29)	NSAIDs	4	OR = 0.51	0.30 to 0.88	0.02	49%	NR	Dyspepsia, nausea/vomiting, loose stool, constipation, abdominal pain/distension and gastrointestinal bleeding/melena
Zhao et al. (31)	NSAIDs	20	OR = 0.51	0.25 to 0.94	NR	NR	Susp. (F)	NR
Hidayat et al. (32)	NR	7	NR	NR	NR	NR	NR	Abdominal discomforts, diarrhea, dyspepsia, mild gastrointestinal symptoms, nausea, restlessness and tingling sensation
Wai et al. (35)	NR	5	NR	NR	NR	NR	NR	Gastrointestinal symptoms, respiratory, neurological and sensory symptoms, dermatological reactions, cardiovascular and edema, metabolic effects and weight changes, general body pain, complications and medical problems and miscellaneous

^a^Publication bias: NS indicates not significant; (E) indicates Egger’s test. Susp. (F) indicates suspected publication bias based on funnel plot inspection; Not Susp. (F) indicates that publication bias is not suspected based on funnel plot inspection; NR indicates not reported. Underlined values are significant.

Pooled analyses showed no statistically significant increase in adverse events with curcumin versus placebo. For example, Onakpoya et al. (36) reported RR = 1.26 (95% CI: 0.56–2.84) for gastrointestinal symptoms; Zeng et al. (30) reported RR = 1.18 (95% CI: 0.71–1.94) for diarrhea; Feng et al. (28) reported RR = 1.07 (95% CI: 0.70–1.65) for composite gastrointestinal symptoms (I^2^ = 0%–32.6%).

Compared with NSAIDs, curcumin showed a more favorable safety profile. Zeng et al. (30) reported RR = 0.55 (95% CI: 0.34–0.88, *p* = 0.01, I^2^ = 70%). Feng et al. (28) reported RR = 0.65 (95% CI: 0.41–1.03, *p* = 0.065), which became significant after excluding one study (RR = 0.63, 95% CI: 0.41–0.95, *p* = 0.026). Wan et al. (29), Zhao et al. (31) both reported OR = 0.51 (95% CI: 0.30–0.88 and 0.25–0.94, respectively).

Heterogeneity I^2^ ranged from 0% to 70%. Publication bias was assessed in a subset of the meta-analyses. Feng et al. (28) noted funnel plot asymmetry (Egger *p* = 0.179).

#### 3.4.3 Exploratory analysis: the influence of curcumin preparation categories

An exploratory analysis was performed to characterize the spectrum of curcumin formulations examined in the systematic review. Given the absence of meta-analytic comparisons between distinct formulation categories, this section presents a descriptive synthesis of the evaluated formulation types and their representation within the evidence base. The distribution of these categories across the 10 included studies is presented in Table 6 (28–37).

**TABLE 6:** Curcumin formulation categories reported in included meta-analyses.

References	Formulation categories included	Category 1: standardized curcuminoids	Category 2: enhanced bioavailability formulations	Category 3: turmeric/raw extracts
Igho et al. (36)	Category 1 + 2 + 3	Curcuminoids	Theracurmin^®^	*C. longa* extract, *C. domestica* extracts
Paultre et al. (37)	Category 1 + 2	Curcuminoids, Curcumin extract	BCM-95^®^, Theracurmin^®^, Turmacin^®^, Curene^®^	None
Shokri-Mashhadi et al. (33)	Category 1 + 2 + 3	Curcuminoids	Meriva^®^, BCM-95^®^, Theracurmin^®^, Nanocurcumin, SLCP	*Curcuma longa* extract, *C. domestica* extract
Zeng et al. (30)	Category 1 + 3	Curcuminoids (C3 Complex^®^), SinaCurcumin	BCM-95^®^	*Curcuma longa* extract
Feng et al. (28)	Category 1 + 2 + 3	Curcuminoids (C3 Complex^®^, CuraMed^®^, Curene^®^)	Theracurmin^®^, BCM-95^®^, Longvida^®^, Nano-curcumin (SinaCurcumin^®^)	*Curcuma longa* extract (Haridra^®^, Turmacin Plus)
Gutiérrez Ruiz et al. (34)	Category 1 + 3	Curcumin	None	*Curcuma longa* extract, Curcuma extract
Wan et al. (29)	Category 1 + 2 + 3	Curcuminoid (*C. domestica*), Curcuminoids (CGM)	BCM-95^®^, SLCP	*Curcuma longa* extract (SinaCurcumin^®^, NR-INF-02)
Jinlong et al. (31)	Category 1 + 2 + 3	Curcumin, Curcuminoids, Curcuminoid complex extract	None	*Curcuma longa* extract, *C. domestica* extract
Hidayat et al. (32)	Category 3	None	None	*Curcuma longa* extract
Wai et al. (35)	Category 1 + 2	Curcuminoids, C3 Complex^®^	BCM-95^®^ (CuraMed^®^), Theracurmin^®^, Longvida^®^, SinaCurcumin^®^, Curene^®^, Curcugen^®^	*C. longa* extract (Haridra), *C. domestica* extract

Category assignment followed the criteria defined in Methods (section “2.4 Intervention”): Category 1, no explicit bioavailability-enhancing technology; Category 2, explicit use of bioavailability-enhancing technologies; Category 3, whole turmeric or non-standardized extracts.

The majority of systematic reviews (6/10) incorporated evidence spanning all three formulation categories (conventional, bioavailability-enhanced, and whole turmeric/non-standardized extracts), thereby capturing the breadth of available literature. Only one review [Hidayat et al. (32)] exclusively examined Category 3 formulations, while two reviews [Paultre et al. (37); Su Wai et al. (35)] restricted their analyses to Categories 1 and 2.

This preliminary assessment confirms substantial heterogeneity in the curcumin formulations investigated for OA. This variability encompasses fundamental compositional differences—ranging from crude plant extracts to purified curcuminoid preparations—as well as technological distinctions, from basic extracts to sophisticated delivery systems engineered to enhance bioavailability. While this diversity reflects the field’s evolution, it also represents a key source of clinical and methodological heterogeneity, potentially confounding pooled outcome interpretations. Notably, the lack of direct comparative meta-analyses precludes definitive conclusions regarding the relative efficacy of different formulation categories. As discussed in Section “3.4.1 Therapeutic effect: the overall effect of curcumin on pain and function,” consistent therapeutic signals for pain relief and functional improvement were observed across all formulation types.

In summary, this analysis demonstrates that OA research has evaluated a wide array of curcumin formulations, with most systematic reviews encompassing the full spectrum. Current evidence suggests potential efficacy across diverse formulations, though no clear superiority hierarchy has been established. Future investigations should prioritize direct comparative trials or network meta-analyses to determine which curcumin formulation optimally balances efficacy and safety in OA management.

### 3.5 Assessment of overlap of primary studies

A total of 38 unique primary RCTs were identified across the 10 included systematic reviews. The total number of citations (i.e., the sum of occurrences of each primary RCT across reviews) was *N* = 133. Using the CCA formula, we obtained a CCA of 27.8%, which falls into the high overlap category.

This high overlap indicates that the same set of primary RCTs has been repeatedly included across multiple systematic reviews. Consequently, the number of systematic reviews (c = 10) does not equate to independent evidence bodies. This finding further supports our decision to avoid quantitative meta-analysis at the umbrella level and to rely on a qualitative synthesis with careful interpretation.

## 4 Discussion

This umbrella review systematically assessed the clinical efficacy of diverse curcumin formulations in OA management by synthesizing evidence from 10 meta-analyses and systematic reviews. The results indicate that curcumin exhibits efficacy signals for pain alleviation and functional improvement, alongside signals of better tolerability relative to NSAIDs in the included studies, though adverse event definitions and reporting completeness varied considerably across reviews. However, substantial heterogeneity was observed across studies, largely attributable to variations in formulation methodologies. Due to the absence of direct comparative analyses among formulations in existing literature, this study employed a qualitative evidence synthesis approach rather than quantitative meta-analysis. While the qualitative assessment did not yield definitive efficacy rankings, it underscores the pivotal role of formulation type in determining clinical outcomes, necessitating careful consideration in both research and clinical applications.

The heterogeneity observed in this umbrella review warrants detailed examination. Beyond formulation differences, this heterogeneity encompasses three interconnected forms: clinical, methodological, and statistical. Clinical heterogeneity arises from variations in patient populations, including OA severity, affected joint sites (knee, hip, hand), baseline pain levels, and concurrent comorbidities (e.g., obesity, diabetes, hypertension). Methodological heterogeneity stems from differences in trial design, such as blinding procedures (double-blind vs. single-blind), allocation concealment, outcome assessment tools (different versions of VAS or WOMAC scales), placebo compositions, and follow-up durations. Statistical heterogeneity, quantified by I^2^ up to 99%, reflects the cumulative impact of the above variations, as well as the pooling of effect sizes across diverse formulations (conventional vs. bioavailability-enhanced vs. whole extracts), dosages (450–2,000 mg daily), and treatment durations (5–11 weeks). The extreme statistical inconsistency (I^2^ up to 99%) reflects the cumulative impact of these factors, which substantially limits the external validity of any single pooled effect estimate. In practice, this means that a pooled estimate derived from such heterogeneous data may not be directly applicable to a specific patient subgroup or a particular curcumin formulation. For instance, an effect size calculated from trials predominantly involving mild knee OA with short-term use of nano-formulated curcumin cannot be reliably generalized to patients with severe hip OA receiving conventional extracts. Therefore, the most robust conclusion from the current evidence is the consistent direction of effect—curcumin provides meaningful pain relief and functional improvement with a favorable safety profile—rather than a precise, generalizable magnitude of effect. Despite the qualitative nature of this synthesis, the consistency of findings across multiple independent meta-analyses—including those rated as high or moderate quality by AMSTAR-2—reinforces the robustness of this directional conclusion.

We further assessed the degree of overlap of primary RCTs across the 10 included systematic reviews using the CCA method. The CCA was 27.8%, indicating high overlap. This means that the same set of primary RCTs has been repeatedly included across multiple meta-analyses, and the number of systematic reviews does not equate to independent evidence bodies. Therefore, interpreting the consistency of findings across reviews as independent confirmation would be inappropriate. The observed consistency remains supportive but should be viewed as aggregated signals from a shared evidence pool rather than replicated validation. While this does not invalidate our qualitative conclusions — the consistent direction of effect across overlapping reviews remains meaningful — it does suggest that some of the apparent consistency may partly reflect repeated analysis of the same underlying data rather than truly independent replication. This finding further supports our decision to avoid quantitative meta-analysis at the umbrella level and reinforces the need for future primary research to conduct new, large-scale, head-to-head RCTs rather than additional meta-analyses of the same small pool of trials.

These findings corroborate a prior umbrella review (38) that quantitatively confirmed the overall efficacy of curcuminoids for OA. However, that study did not focus on formulation-dependent effects, nor did it examine how formulation heterogeneity might influence pooled estimates or obscure differential effectiveness among preparation types. Additionally, the prior review did not systematically categorize the sources of heterogeneity into clinical, methodological, and statistical components, nor did it discuss their implications for external validity. It also did not include the most recent evidence (published up to 2025) or explore the distribution of different formulation categories across the included studies. To address this evidence gap, the present review provides a qualitative synthesis organized specifically by formulation categories, assessing the heterogeneity introduced by formulation diversity and examining its implications for external validity. Owing to substantial clinical and methodological heterogeneity that precluded quantitative pooling, we adopted a narrative approach supported by detailed summary tables that comprehensively characterize the included systematic reviews, including the distribution of formulation categories across studies and the latest evidence up to 2025. This structured presentation is intended to derive clinically relevant insights and to inform future research priorities, including specific suggestions for head-to-head trial designs. Across formulations, curcumin demonstrated statistically significant improvements in both VAS pain scores and WOMAC composite scores relative to placebo. These favorable outcomes were consistently replicated in multiple independent meta-analyses, reinforcing curcumin’s potential as a viable therapeutic option for OA with clinically meaningful symptom mitigation.

Notably, this study provides a comprehensive evaluation of formulation-specific effects. Although direct comparative analysis remains methodologically challenging, qualitative synthesis reveals that all three formulation categories—conventional curcumin (Category 1), bioavailability-enhanced curcumin (Category 2, e.g., phospholipid complexes, nano-formulations, piperine combinations), and whole turmeric/non-standardized extracts (Category 3)—exhibit therapeutic efficacy comparable to NSAIDs while maintaining enhanced safety profiles. While the study does not establish a definitive hierarchy among formulations, Table 6 suggests that bioavailability-enhanced formulations may represent a predominant trend in future development. The majority of included studies focused on this category, which, despite being less extensively investigated than conventional formulations, has gained prominence through ongoing optimization of systemic delivery mechanisms. Nevertheless, these conclusions warrant further validation via rigorous, design-focused research to substantiate their clinical relevance.

The current investigation yielded clinically significant and robust findings regarding tolerability outcomes. Systematic qualitative analysis consistently revealed favorable tolerability profiles across all curcumin formulation categories. The observed incidence of adverse events—predominantly gastrointestinal reactions—demonstrated no statistically significant difference compared to placebo controls. Notably, curcumin formulations appeared to have a lower adverse event risk relative to NSAIDs in the available studies, suggesting a therapeutic advantage in terms of tolerability. It is important to note, however, that adverse event definitions were not uniform across the included systematic reviews, and several primary RCTs lacked systematic safety reporting. Therefore, while curcumin shows signals of better tolerability relative to NSAIDs, this should be interpreted as a favorable tolerability profile in the available evidence rather than a definitive safety advantage. These tolerability signals, together with efficacy signals comparable to NSAIDs, suggest that curcumin formulations may be suitable for long-term management of chronic OA, especially for patients who require sustained symptom control but have contraindications or intolerance to conventional NSAIDs.

Several limitations warrant consideration. First, the absence of direct comparative trials precludes definitive hierarchical ranking of formulation categories. Existing meta-analyses either aggregate diverse formulations or perform subgroup analyses without rigorous formulation-specific categorization, creating a paradox: although curcumin is clearly efficacious and demonstrates that advanced formulations overcome bioavailability issues, evidence on optimal administration remains insufficient. This gap is aggravated by inconsistent primary reporting. For instance, many trials only describe “curcumin extract” without specifying concentration, extraction method, or bioavailability-enhancing technology, making category assignment impossible (39). Others report “nanocurcumin” but omit critical parameters such as particle size, polydispersity index, or encapsulation efficiency (40). Such omissions hinder accurate classification and obscure clinically relevant pharmacokinetic differences, so pooled estimates may conceal within-group heterogeneity and dilute true differential effects across categories. Second, the available evidence permits only qualitative synthesis. Methodological quality varies substantially: several included studies are low or very low according to AMSTAR-2, with only three rated as high quality, which may compromise estimate reliability. Third, *post hoc* categorization based on published descriptions could introduce misclassification bias; to minimize this risk, three reviewers (two independent assigners and a third arbiter) applied predefined criteria.

Addressing these limitations, future investigations should prioritize several directions. First, well-designed RCTs with head-to-head comparisons between clearly defined formulation categories are urgently needed A. pragmatic three-arm design could compare a representative conventional extract (Category 1), a leading bioavailability-enhanced formulation (Category 2), and a whole turmeric extract (Category 3), using a non-inferiority design against an active comparator such as ibuprofen at standard analgesic doses. Co-primary outcomes might include change in VAS pain and WOMAC function score at 8–12 weeks; secondary outcomes could encompass responder rates, rescue medication use, and adverse events. Such trials face several non-trivial challenges: blinding is complicated by differing capsule appearances or potential taste differences of turmeric extracts; standardization of active ingredients across complex natural products (curcuminoid content, piperine concentration, particle size) requires rigorous quality control; and the costs of manufacturing and testing multiple active formulations are substantial. Collaborative efforts among academic centers, industry sponsors, and regulatory bodies will be essential to overcome these hurdles. Second, primary studies must adopt standardized reporting of formulation technologies (e.g., particle size, encapsulation efficiency), excipient composition, and curcuminoid content to enable accurate classification and cross-trial comparability. Third, systematic reviews and meta-analyses should move beyond aggregated analyses and conduct rigorous formulation-stratified subgroup analyses or network meta-analyses to indirectly compare different categories. Fourth, a more precise and detailed classification system for curcumin preparations—potentially including subcategories based on specific bioavailability-enhancing mechanisms—should be developed and validated to facilitate robust comparative analyses. Implementing these recommendations will help translate the promising but fragmented current evidence into clinically actionable guidance for curcumin-based OA interventions.

## 5 Conclusion

This umbrella review provides qualitative evidence that curcumin shows efficacy signals comparable to NSAIDs for OA symptom mitigation and appears to have a more favorable tolerability profile in the available studies, although safety data were reported inconsistently across primary studies. However, substantial heterogeneity and the absence of head-to-head formulation comparisons preclude definitive claims of superiority or formulation-specific ranking. These findings should be interpreted with caution, but they support curcumin as a promising option in OA management. Nevertheless, despite promising findings, substantial heterogeneity among studies and the paucity of robust comparative clinical trials remain significant limitations, precluding definitive conclusions regarding optimal formulation strategies. Consequently, clinical implementation warrants judicious consideration. Although curcumin presents notable adjunctive potential in OA management, its therapeutic application should be informed by thorough characterization of formulation-specific pharmacokinetic properties. Clinicians are advised to select preparations with well-documented bioavailability-enhancing mechanisms. To fully harness curcumin’s therapeutic potential in OA, future investigations should transition from efficacy validation to systematic comparative evaluation of different formulation approaches in clinical contexts, thereby strengthening the evidence base for curcumin-based OA interventions.

## Data Availability

The original contributions presented in this study are included in this article/Supplementary material, further inquiries can be directed to the corresponding author.

## References

[1] Xia B Di C Zhang J Hu S Jin H Tong P. Osteoarthritis pathogenesis: a review of molecular mechanisms. *Calcif Tissue Int.* (2014) 95:495–505. 10.1007/s00223-014-9917-9 25311420 PMC4747051

[2] Fang Y Wang P Xia L Bai S Shen Y Li Q et al. Aberrantly hydroxymethylated differentially expressed genes and the associated protein pathways in osteoarthritis. *PeerJ.* (2019) 7:e6425. 10.7717/peerj.6425 30828485 PMC6394344

[3] Iantomasi T Aurilia C Donati S Falsetti I Palmini G Carossino R et al. Oxidative stress, micrornas, and long non-coding rnas in osteoarthritis pathogenesis: cross-talk and molecular mechanisms involved. *Int J Mol Sci.* (2025) 26:6428. 10.3390/ijms26136428 40650204 PMC12250001

[4] Tan C Zhang J Chen W Feng F Yu C Lu X et al. Inflammatory cytokines via up-regulation of aquaporins deteriorated the pathogenesis of early osteoarthritis. *PLoS One.* (2019) 14:e0220846. 10.1371/journal.pone.0220846 31404098 PMC6690536

[5] Terkawi MA Ebata T Yokota S Takahashi D Endo T Matsumae G et al. Low-Grade inflammation in the pathogenesis of osteoarthritis: cellular and molecular mechanisms and strategies for future therapeutic intervention. *Biomedicines.* (2022) 10:1109. 10.3390/biomedicines10051109 35625846 PMC9139060

[6] Li L Sun Y Luo J Liu M. Circulating immune cells and risk of osteosarcoma: a mendelian randomization analysis. *Front Immunol.* (2024) 15:1381212. 10.3389/fimmu.2024.1381212 39081321 PMC11286390

[7] Peng Z Huang W Tang M Chen B Yang R Liu Q et al. Investigating the shared genetic architecture between hypothyroidism and rheumatoid arthritis. *Front Immunol.* (2024) 14:1286491. 10.3389/fimmu.2023.1286491 38332917 PMC10850220

[8] Poole AR. Current opinion: where are we in our understanding and treatment of osteoarthritis? *Swiss Med Wkly.* (2016) 146:w14340. 10.4414/smw.2016.14340 27463798

[9] Yang Z Mathieson S Kobayashi S Abdel Shaheed C Nogueira LAC Simic M et al. Prevalence of nonsteroidal antiinflammatory drugs prescribed for osteoarthritis: a systematic review and meta-analysis of observational studies. *Arthritis Care Res.* (2023) 75:2345–58. 10.1002/acr.25157 37221152

[10] Gehling M Hermann B Tryba M. Meta-Analysis of dropout rates in randomized controlled clinical trials: opioid analgesia for osteoarthritis pain. *Schmerz.* (2011) 25:296–305. 10.1007/s00482-011-1057-9 21614601

[11] Drummer D McAdam J Seay R Ferrando A Bridges SL Jr. Singh JA et al. Osteoarthritis progression: mitigation and rehabilitation strategies. *Front Rehabil Sci.* (2021) 2:724052. 10.3389/fresc.2021.724052 36188773 PMC9397730

[12] Swallow J Seidler K Barrow M. The mechanistic role of curcumin on matrix metalloproteinases in osteoarthritis. *Fitoterapia.* (2024) 174:105870. 10.1016/j.fitote.2024.105870 38423225

[13] Pan Y Liu X Wang S Chen L Li A Chi Q et al. Coniferyl aldehyde in ginger-eucommiae cortex enhances osteoarthritis treatment by modulating aldoa and H3k23la histone lactylation. *Phytomedicine.* (2026) 152:157852. 10.1016/j.phymed.2026.157852 41619548

[14] Zazueta-Beltrán L Medina-Aymerich L Estela Díaz-Triste N Chávez-Piña AE Castañeda-Hernández G Cruz-Antonio L. Evidence against the participation of a pharmacokinetic interaction in the protective effect of single-dose curcumin against gastrointestinal damage induced by indomethacin in rats. *J Integr Med.* (2017) 15:151–7. 10.1016/s2095-4964(17)60324-8 28285620

[15] Tsuda T. Curcumin as a functional food-derived factor: degradation products, metabolites, bioactivity, and future perspectives. *Food Funct.* (2018) 9:705–14. 10.1039/c7fo01242j 29206254

[16] Obeid MA Alsaadi M Aljabali AA. Recent updates in curcumin delivery. *J Liposome Res.* (2023) 33:53–64. 10.1080/08982104.2022.2086567 35699160

[17] Mor N Raghav N. Alginate hydrogels: sustained release system to analyze the effect of traditional excipients on curcumin availability. *Bioorg Chem.* (2021) 107:104513. 10.1016/j.bioorg.2020.104513 33279244

[18] Song IS Cha JS Choi MK. Characterization, in vivo and in vitro evaluation of solid dispersion of curcumin containing D-α-Tocopheryl polyethylene glycol 1000 succinate and mannitol. *Molecules.* (2016) 21:1386. 10.3390/molecules21101386 27763524 PMC6274229

[19] Purpura M Lowery RP Wilson JM Mannan H Münch G Razmovski-Naumovski V. Analysis of different innovative formulations of curcumin for improved relative oral bioavailability in human subjects. *Eur J Nutr.* (2018) 57:929–38. 10.1007/s00394-016-1376-9 28204880 PMC5861163

[20] Ataei M Gumpricht E Kesharwani P Jamialahmadi T Sahebkar A. Recent advances in curcumin-based nanoformulations in diabetes. *J Drug Target.* (2023) 31:671–84. 10.1080/1061186x.2023.2229961 37354074

[21] Rahimi HR Nedaeinia R Sepehri Shamloo A Nikdoust S Kazemi Oskuee R. Novel delivery system for natural products: nano-curcumin formulations. *Avicenna J Phytomed.* (2016) 6:383–98. 27516979 PMC4967834

[22] Zhong D Xie W Liao Z Liu H Zhu L Gao F et al. Ythdf1 transcriptionally activated by Tcf4 suppresses osteoblast ferroptosis in titanium nanoparticle-induced osteolysis by accelerating Gpx4 and Slc7a11 translation. *J Nanobiotechnol.* (2025) 23:783. 10.1186/s12951-025-03861-6 41430267 PMC12723855

[23] Chen D Liu Y Fan KY Xie YQ Yu AA Xia ZH et al. [Relation between drug release and the drug status within curcumin-loaded microsphere]. *Yao Xue Xue Bao.* (2016) 51:140–6. 27405176

[24] Shehata TM Ibrahim MM Elsewedy HS. Curcumin niosomes prepared from proniosomal gels: in vitro skin permeability, kinetic and in vivo studies. *Polymers* (2021) 13:791. 10.3390/polym13050791 33806659 PMC7961916

[25] Moher D Shamseer L Clarke M Ghersi D Liberati A Petticrew M et al. Preferred reporting items for systematic review and meta-analysis protocols (Prisma-P) 2015 statement. *Syst Rev.* (2015) 4:1. 10.1186/2046-4053-4-1 25554246 PMC4320440

[26] Shea BJ Reeves BC Wells G Thuku M Hamel C Moran J et al. Amstar 2: a critical appraisal tool for systematic reviews that include randomised or non-randomised studies of healthcare interventions, or both. *BMJ.* (2017) 358:j4008. 10.1136/bmj.j4008 28935701 PMC5833365

[27] Pieper D Antoine S-L Mathes T Neugebauer EAM Eikermann M. Systematic review finds overlapping reviews were not mentioned in every other overview. *J Clin Epidemiol.* (2014) 67:368–75. 10.1016/j.jclinepi.2013.11.007 24581293

[28] Feng J Li Z Tian L Mu P Hu Y Xiong F et al. Efficacy and safety of curcuminoids alone in alleviating pain and dysfunction for knee osteoarthritis: a systematic review and meta-analysis of randomized controlled trials. *BMC Complement Med Ther.* (2022) 22:276. 10.1186/s12906-022-03740-9 36261810 PMC9580113

[29] Wan Y Sun W Yang J Ren J Kou Q. The comparison of curcuminoid formulations or its combination with conventional therapies versus conventional therapies alone for knee osteoarthritis. *Clin Rheumatol.* (2022) 41:2153–69. 10.1007/s10067-022-06105-2 35294665

[30] Zeng L Yu G Hao W Yang K Chen H. The efficacy and safety of curcuma longa extract and curcumin supplements on osteoarthritis: a systematic review and meta-analysis. *Biosci Rep.* (2021) 41:BSR20210817. 10.1042/bsr20210817 34017975 PMC8202067

[31] Zhao J Liang G Zhou G Hong K Yang W Liu J et al. Efficacy and safety of curcumin therapy for knee osteoarthritis: a bayesian network meta-analysis. *J Ethnopharmacol.* (2024) 321:117493. 10.1016/j.jep.2023.117493 38036015

[32] Hidayat R Parlindungan F Nisa JI Mahendra AI Indika MI Efendi C. Efficacy of curcuma longa in relieving pain symptoms of knee osteoarthritis patients: a systematic review and meta-analysis of clinical trials. *J Rheum Dis.* (2025) 32:17–29. 10.4078/jrd.2024.0062 39712249 PMC11659657

[33] Shokri-Mashhadi N Bagherniya M Askari G Sathyapalan T Sahebkar AA. Systematic review of the clinical use of curcumin for the treatment of osteoarthritis. *Adv Exp Med Biol.* (2021) 1291:265–82. 10.1007/978-3-030-56153-6_16 34331696

[34] Gutierrez Ruiz A San Mauro Martin I Garicano Vilar E Brata C Camina Martin MA. Efficacy and safety of curcuma longa extract as a treatment of primary knee osteoarthritis in adults and elderly: a systematic review. *Prog Nutr.* (2022) 24:e2022026. 10.23751/pn.v24i1.11651

[35] Wai HS Pathomwichaiwat T Suansanae T Nathisuwan S Rattanavipanon W. Effect of turmeric products on knee osteoarthritis: a systematic review and network meta-analysis. *BMC Complement Med Ther.* (2025) 25:292. 10.1186/s12906-025-05045-z 40731001 PMC12309109

[36] Onakpoya IJ Spencer EA Perera R Heneghan CJ. Effectiveness of curcuminoids in the treatment of knee osteoarthritis: a systematic review and meta-analysis of randomized clinical trials. *Int J Rheum Dis.* (2017) 20:420–33. 10.1111/1756-185x.13069 28470851

[37] Paultre K Cade W Hernandez D Reynolds J Greif D Best TM. Therapeutic effects of turmeric or curcumin extract on pain and function for individuals with knee osteoarthritis: a systematic review. *BMJ Open Sport Exerc Med.* (2021) 7:e000935. 10.1136/bmjsem-2020-000935 33500785 PMC7812094

[38] Bideshki MV Jourabchi-Ghadim N Radkhah N Behzadi M Asemani S Jamilian P et al. The efficacy of curcumin in relieving osteoarthritis: a meta-analysis of meta-analyses. *Phytother Res.* (2024) 38:2875–91. 10.1002/ptr.8153 38576215

[39] Popovic VB Farhat EK Banjari I Kadic AJ Puljak L. Bioavailability of oral curcumin in systematic reviews: a methodological study. *Pharmaceuticals.* (2024) 17:164. 10.3390/ph17020164 38399379 PMC10891944

[40] Naksuriya O Okonogi S Schiffelers RM Hennink WE. Curcumin nanoformulations: a review of pharmaceutical properties and preclinical studies and clinical data related to cancer treatment. *Biomaterials.* (2014) 35:3365–83. 10.1016/j.biomaterials.2013.12.090 24439402

