# Supplementary material for: Efficacy and safety of different curcumin formulations in osteoarthritis: an umbrella review of systematic reviews
Source: Front Med (Lausanne). 2026 May 21;13:1801273. doi: 10.3389/fmed.2026.1801273 (PMC13233388; doi:10.3389/fmed.2026.1801273)
Supplement: Supplementary file 1 [file Table_1.docx]

1. PubMed：

- (((((((((((((((((((Osteoarthritis[MeSH Terms]) OR (Osteoarthritis)) OR (Osteoarthritides)) OR (Arthritis, Degenerative)) OR (Arthritides, Degenerative)) OR (Degenerative Arthritides)) OR (Degenerative Arthritis)) OR (Osteoarthrosis)) OR (Osteoarthroses)) OR (Osteoarthrosis Deformans)) OR (Arthrosis)) OR (Arthroses)) OR (arthritis, noninflammatory)) OR (degenerative joint disease)) OR (noninflammatory arthritis)) OR (osteo-arthritis)) OR (primary osteoarthritis)) OR (rheumatoid arthrosis)) AND (((((((((((((Curcumin[MeSH Terms]) OR (Curcumin)) OR (Curcumin Phytosome)) OR (Phytosome, Curcumin)) OR (1,6-Heptadiene-3,5-dione, 1,7-bis(4-hydroxy-3-methoxyphenyl)-, (E,E)-)) OR (Diferuloylmethane)) OR (Turmeric Yellow)) OR (Yellow, Turmeric)) OR (Mervia)) OR (1, 7 bis (4 hydroxy 3 methoxyphenyl) 1, 6 heptadiene 3, 5 dione)) OR (bis (4 hydroxy 3 methoxycinnamoyl) methane)) OR (curcumine)) OR (nanocurc))) AND (((((((((meta-analysis[MeSH Terms])) OR (meta-analysis)) OR (systematic review)) OR (meta analysis)) OR (meta review)) OR (meta)) OR (metaanalysis)) OR (analysis, meta))

1. Web of science：

- (TS=(Osteoarthritis) OR TS=(Osteoarthritides) OR TS=(Arthritis, Degenerative) OR TS=(Arthritides, Degenerative) OR TS=(Degenerative Arthritides) OR TS=(Degenerative Arthritis) OR TS=(Osteoarthrosis) OR TS=(Osteoarthroses) OR TS=(Osteoarthrosis Deformans) OR TS=(Arthrosis) OR TS=(Arthroses) OR TS=(arthritis, noninflammatory) OR TS=(degenerative joint disease) OR TS=(noninflammatory arthritis) OR TS=(osteo-arthritis) OR TS=(primary osteoarthritis) OR TS=(rheumatoid arthrosis)) NOT (SILOID==("PPRN") OR SILOID==("RC"))
- (TS=(Curcumin) OR TS=(Curcumin Phytosome) OR TS=(Phytosome, Curcumin) OR TS=(1,6-Heptadiene-3,5-dione, 1,7-bis(4-hydroxy-3-methoxyphenyl)-, (E,E)-) OR TS=(Diferuloylmethane) OR TS=(Turmeric Yellow) OR TS=(Yellow, Turmeric) OR TS=(Mervia) OR TS=(1, 7 bis (4 hydroxy 3 methoxyphenyl) 1, 6 heptadiene 3, 5 dione) OR TS=(bis (4 hydroxy 3 methoxycinnamoyl) methane) OR TS=(curcumine) OR TS=(nanocurc)) NOT (SILOID==("PPRN") OR SILOID==("RC"))
- (TS=(meta-analysis) OR TS=(systematic review) OR TS=(meta analysis) OR TS=(meta review) OR TS=(meta) OR TS=(metaanalysis) OR TS=(analysis, meta)) NOT (SILOID==("PPRN") OR SILOID==("RC"))
- (#1 AND #2 AND #3) NOT (SILOID==("PPRN") OR SILOID==("RC"))

1. Cochrance：

- MeSH descriptor: [Osteoarthritis] explode all trees
- MeSH descriptor: [Curcumin] explode all trees
- (Osteoarthritides):ti,ab,kw OR (Arthritis, Degenerative):ti,ab,kw OR (Arthritides, Degenerative):ti,ab,kw OR (Degenerative Arthritides):ti,ab,kw OR (Degenerative Arthritis):ti,ab,kw
- (Osteoarthrosis):ti,ab,kw OR (Osteoarthroses):ti,ab,kw OR (Osteoarthrosis Deformans):ti,ab,kw OR (Arthrosis):ti,ab,kw OR (Arthroses):ti,ab,kw
- (arthritis, noninflammatory):ti,ab,kw OR (degenerative joint disease):ti,ab,kw OR (noninflammatory arthritis):ti,ab,kw OR (osteo-arthritis):ti,ab,kw OR (primary osteoarthritis):ti,ab,kw
- (rheumatoid arthrosis):ti,ab,kw
- #1 OR #3 OR #4 OR #5 OR #6
- (Yellow, Turmeric):ti,ab,kw OR (Mervia):ti,ab,kw OR (1, 7 bis (4 hydroxy 3 methoxyphenyl) 1, 6 heptadiene 3, 5 dione):ti,ab,kw OR (bis (4 hydroxy 3 methoxycinnamoyl) methane):ti,ab,kw OR (curcumine):ti,ab,kw
- (Curcumin Phytosome):ti,ab,kw OR (Phytosome, Curcumin):ti,ab,kw OR (Diferuloylmethane):ti,ab,kw OR (Turmeric Yellow):ti,ab,kw OR (nanocurc):ti,ab,kw
- #2 OR #8 OR #9
- (meta-analysis):ti,ab,kw OR (systematic review):ti,ab,kw OR (meta analysis):ti,ab,kw OR (meta review):ti,ab,kw OR (meta):ti,ab,kw
- (analysis, meta):ti,ab,kw OR (metaanalysis):ti,ab,kw
- #11 OR #12
- #7 AND #10 AND #13

1. Embase：

- #3 AND #6 AND #9
- #7 OR #8
- 'meta analysis':ab,ti OR 'systematic review':ab,ti OR 'meta-analysis':ab,ti OR 'meta review':ab,ti OR meta:ab,ti OR 'analysis, meta':ab,ti OR metaanalysis:ab,ti
- 'meta analysis'/exp
- #4 OR #5
- 'curcumin':ab,ti OR 'curcumin phytosome':ab,ti OR 'phytosome, curcumin':ab,ti OR curcumine:ab,ti OR diferuloylmethane:ab,ti OR 'turmeric yellow':ab,ti OR 'yellow, turmeric':ab,ti OR mervia:ab,ti OR nanocurc:ab,ti OR ('1, 7 bis':ab,ti AND '4 hydroxy 3 methoxyphenyl':ab,ti AND '1, 6 heptadiene 3, 5 dione':ab,ti) OR (bis:ab,ti AND '4 hydroxy 3 methoxycinnamoyl':ab,ti AND methane:ab,ti)
- 'curcumin'/exp
- #1 OR #2
- 'osteoarthritis':ab,ti OR osteoarthritides:ab,ti OR 'arthritis, degenerative':ab,ti OR 'arthritides, degenerative':ab,ti OR 'degenerative arthritides':ab,ti OR 'degenerative arthritis':ab,ti OR osteoarthrosis:ab,ti OR osteoarthroses:ab,ti OR 'osteoarthrosis deformans':ab,ti OR arthrosis:ab,ti OR arthroses:ab,ti OR 'arthritis, noninflammatory':ab,ti OR 'degenerative joint disease':ab,ti OR 'noninflammatory arthritis':ab,ti OR 'osteo arthritis':ab,ti OR 'primary osteoarthritis':ab,ti OR 'rheumatoid arthrosis':ab,ti
- 'osteoarthritis'/exp

1. Scopus：

- ( ( TITLE-ABS-KEY ( Osteoarthritis ) OR TITLE-ABS-KEY ( Osteoarthritides ) OR TITLE-ABS-KEY ( Arthritis , Degenerative ) OR TITLE-ABS-KEY ( Arthritides , Degenerative ) OR TITLE-ABS-KEY ( Degenerative Arthritides ) OR TITLE-ABS-KEY ( Degenerative Arthritis ) OR TITLE-ABS-KEY ( Osteoarthrosis ) OR TITLE-ABS-KEY ( Osteoarthroses ) OR TITLE-ABS-KEY ( Osteoarthrosis Deformans ) OR TITLE-ABS-KEY ( Arthrosis ) OR TITLE-ABS-KEY ( Arthroses ) AND TITLE-ABS-KEY ( arthritis , noninflammatory ) OR TITLE-ABS-KEY ( degenerative joint disease ) OR TITLE-ABS-KEY ( noninflammatory arthritis ) OR TITLE-ABS-KEY ( osteo-arthritis ) OR TITLE-ABS-KEY ( primary osteoarthritis ) OR TITLE-ABS-KEY ( rheumatoid arthrosis ) ) ) AND ( ( TITLE-ABS-KEY ( Curcumin ) OR TITLE-ABS-KEY ( Curcumin Phytosome ) OR TITLE-ABS-KEY ( Phytosome Curcumin ) OR TITLE-ABS-KEY ( Diferuloylmethane ) OR TITLE-ABS-KEY ( Turmeric Yellow ) OR TITLE-ABS-KEY ( Turmeric Yellow ) OR TITLE-ABS-KEY ( Mervia ) OR TITLE-ABS-KEY ( curcumine ) OR TITLE-ABS-KEY ( nanocurc ) ) ) AND ( ( TITLE-ABS-KEY ( systematic review ) OR TITLE-ABS-KEY ( meta-analysis ) OR TITLE-ABS-KEY ( meta analysis ) OR TITLE-ABS-KEY ( meta review ) OR TITLE-ABS-KEY ( meta ) OR TITLE-ABS-KEY ( metaanalysis ) OR TITLE-ABS-KEY ( analysis , meta ) ) )

1. Medline：

- MHX=(Osteoarthritis) OR TS=(Osteoarthritides) OR TS=(Arthritis, Degenerative) OR TS=(Arthritides, Degenerative) OR TS=(Degenerative Arthritides) OR TS=(Degenerative Arthritis) OR TS=(Osteoarthrosis) OR TS=(Osteoarthroses) OR TS=(Osteoarthrosis Deformans) OR TS=(Arthrosis) OR TS=(Arthroses) OR TS=(arthritis, noninflammatory) OR TS=(degenerative joint disease) OR TS=(noninflammatory arthritis) OR TS=(osteo-arthritis) OR TS=(primary osteoarthritis) OR TS=(rheumatoid arthrosis)
- MHX=(Curcumin) OR TS=(Curcumin Phytosome) OR TS=(Phytosome, Curcumin) OR TS=(1,6-Heptadiene-3,5-dione, 1,7-bis(4-hydroxy-3-methoxyphenyl)-, (E,E)-) OR TS=(Diferuloylmethane) OR TS=(Turmeric Yellow) OR TS=(Mervia) OR TS=(1, 7 bis (4 hydroxy 3 methoxyphenyl) 1, 6 heptadiene 3, 5 dione) OR TS=(bis (4 hydroxy 3 methoxycinnamoyl) methane) OR TS=(curcumine) OR TS=(nanocurc)
- TS=(meta analysis) OR TS=(systematic review) OR TS=(meta-analysis) OR TS=(meta review) OR TS=(meta) OR TS=(metaanalysis) OR TS=(analysis, meta)
- #1 AND #2 AND #3
